# Prediagnostic Prostate-specific Antigen Testing and Clinical Characteristics in Men with Lethal Prostate Cancer

**DOI:** 10.1016/j.euros.2024.02.011

**Published:** 2024-03-04

**Authors:** Markus Arvendell, Lars Björnebo, Martin Eklund, Ugo Giovanni Falagario, Jan Chandra Engel, Olof Akre, Henrik Grönberg, Tobias Nordström, Anna Lantz

**Affiliations:** a Department of Molecular Medicine and Surgery, Karolinska Institutet, Stockholm, Sweden; b Department of Medical Epidemiology and Biostatistics, Karolinska Institutet, Stockholm, Sweden; c Department of Urology, Södersjukhuset, Stockholm, Sweden; d Department of Urology, University of Foggia, Foggia, Italy; e Department of Clinical Sciences at Danderyd Hospital, Karolinska Institutet, Stockholm, Sweden

**Keywords:** Prostate cancer, Diagnosis, Prostate-specific antigen, Prediagnostic testing, Population-based studies

## Abstract

**Background and objective:**

Prostate cancer (PC) is the fifth leading cause of cancer-related mortality in men worldwide. Opportunistic testing with prostate-specific antigen (PSA) has limited impact on PC mortality. Our objective was to assess prediagnostic PSA testing patterns and clinical characteristics at diagnosis in men with lethal PC.

**Methods:**

We conducted a population-based observational study of all men dying from PC in Stockholm County, Sweden, from 2015 to 2019. Data were retrieved from the National Prostate Cancer Register and the Stockholm PSA and Biopsy Register. If the first PSA was registered within 1 yr before diagnosis, men were categorised as PSA naïve. If an elevated PSA level was registered >1 yr before diagnosis without leading to prostate biopsy or repeating PSA within 1 yr, men were categorised as having delayed diagnosis. If a normal PSA level was registered within 5 yr before diagnosis, followed by an elevated PSA level that resulted in PC diagnosis within 1 yr, men were categorised as PSA tested. Clinical characteristics at diagnosis were stratified with D’Amico risk group classification.

**Key findings and limitations:**

Among 1473 men dying from PC, PSA test history was available for 995. Of these men, 60% (*n* = 592) were PSA naïve, 25% (*n* = 250) received delayed diagnosis, and 15% (*n* = 153) were PSA tested. After examining all 1473 men, 25% (*n* = 350) were diagnosed with low- or intermediate-risk cancer, 48% (*n* = 687) with high-risk cancer, and 27% (*n* = 385) with metastatic disease. Limitations include the retrospective design.

**Conclusions and clinical implications:**

Many men with lethal PC lacked PSA testing before diagnosis or had been tested without subsequent follow-up. Nearly half of the study population was diagnosed with high-risk cancer and almost one-third with metastatic disease. These findings suggest further evaluation of the current opportunistic PSA testing approach.

**Patient summary:**

Data from a population-based observational study of men dying from prostate cancer showed that many of them did not undergo either prostate-specific antigen (PSA) testing before diagnosis or subsequent follow-up if tested. These findings implicate deficiencies in the current opportunistic PSA testing approach.

## Take Home Message

Many men with lethal prostate cancer did not undergo prostate-specific antigen (PSA) testing before diagnosis, or if they did, were not subsequently followed up. This could implicate deficiencies in current opportunistic PSA testing strategies.

## 1 Introduction

Prostate cancer (PC) is the second most prevalent malignancy and the fifth leading cause of cancer-related death in men worldwide [1]. The disease often progresses silently, with limited noticeable symptoms in localised cases, emphasising the importance of early detection through alternative means. In the early 1990s, prostate-specific antigen (PSA) testing was introduced in the USA as a tool for early detection of PC [2]. There is evidence from the large randomised European screening trial ERSPC that PSA screening reduces PC mortality rates [3], [4], [5], [6]. Since then, PSA testing has become widespread and has remained unorganised and opportunistic. Intense opportunistic screening reduces PC mortality more effectively than less intense opportunistic testing [7]. However, opportunistic PSA testing has been proved to be inefficient, unequal, and resource intensive, leading to overdiagnosis and overtreatment of clinically insignificant tumours [8], [9]. In addition, conflicting results have emerged from the randomised PLCO trial, indicating no significant difference in PC mortality between men who underwent organised screening and those subjected to opportunistic testing [10], [11]. Consequently, no major guidelines consistently recommend PSA screening for PC [12], [13], [14]. Nevertheless, PC remains a major health concern, and PSA continues to play a crucial role in the diagnostic chain.

There is a lack of population-based studies specifically addressing opportunistic PSA testing in men who have died from PC. Therefore, we aimed to describe prediagnostic PSA testing patterns among men with lethal PC and clinical characteristics at the time of diagnosis within this specific population.

## 2 Patients and methods

This study employed a retrospective, observational, and descriptive design, encompassing all men who died from PC in Stockholm County, Sweden, from 2015 to 2019. The selected 5-yr period aimed to mitigate potential confounding factors related to the onset of the COVID-19 pandemic in 2020. Data were retrieved from the National Prostate Cancer Register (NPCR) and the Stockholm PSA and Biopsy Register (STHLM0). NPCR is a population-based register containing data on tumour characteristics and primary treatment, including near complete data on all diagnosed PC cases in Sweden [15]. STHLM0 is a population-based register containing data on all PSA tests and prostate biopsies in Stockholm County since 2003.

For prediagnostic PSA testing patterns, each PSA test performed in conjunction with a histopathological examination or alone within 5 yr before diagnosis was examined. The STHLM0 database provided access to all PSA tests conducted from 2003 onwards. Therefore, men diagnosed before 2008 were deemed to have missing data. Elevated PSA levels were defined based on national guidelines, that is, ≥3 ng/ml for men aged ≤69 yr, ≥5 ng/ml for men aged 70–80 yr, and ≥7 ng/ml for men aged >80 yr [16]. Men with the first recorded PSA test taken within 1 yr before diagnosis were categorised as PSA naïve. Men with an elevated PSA level recorded >1 yr before diagnosis, without subsequent histopathological examination or PSA monitoring within 1 yr, were categorised as having delayed diagnosis. Men with a nonelevated PSA level within 5 yr before diagnosis, followed by an elevated PSA level that resulted in PC diagnosis within 1 yr, were categorised as PSA tested.

Clinical disease characteristics at diagnosis were determined using the D’Amico risk group classification based on PSA level, Gleason score, and T stage [17]. Low-risk cancer was defined for patients with a PSA level of ≤9.9 ng/ml, a Gleason score of ≤6, and a T stage of <T2b. Intermediate-risk cancer was defined for those with a PSA level of 10–19.9 ng/ml, a Gleason score of 7, and a T stage equal to T2b. High-risk cancer was defined for those with a PSA level of ≥20 ng/ml, a Gleason score of 8–10, and a T stage of >T2b. However, due to limitations in the available T-stage data within NPCR, patients described as having T2 (without further subdivision into T2a, T2b, and T2c) were considered to have missing data, unless PSA level or Gleason score could define them as having high-risk cancer. Patients diagnosed with N1 (lymph node metastasis) or M1 (visceral or skeletal metastasis) were classified as having metastatic disease at diagnosis.

Descriptive data were presented with median and interquartile range (IQR). Survival proportions were displayed with curves representing time to death, and the log-rank test was performed to compare survival times between the groups in the analysis, considering significance at *p* < 0.05. Statistical analyses were performed in R statistical software, version 4.2.3 (R Foundation for Statistical Computing, Vienna, Austria). Ethical approval for the study was obtained from the Swedish Ethical Review Authority (dnr 2021-04497; date of research ethical approval for the study: September 13, 2021).

## 3 Results

From 2015 to 2019, 1473 men died from PC in Stockholm County. Table 1 provides an overview of the clinical disease characteristics at diagnosis. The median age at diagnosis was 73 yr (IQR: 67–79 yr), and the median PSA level at diagnosis was 28 ng/ml (IQR: 11–100 ng/ml). According to D'Amico risk group classification, 5% (*n* = 68) of the patients were diagnosed with low-risk cancer, 20% (*n* = 282) with intermediate-risk cancer, and 48% (*n* = 687) with high-risk cancer. At the time of diagnosis, 27% (*n* = 385) of the patients had metastatic disease. The consequent treatment approach had a curative intent (active treatment) in 315 patients (23%), while the majority (*n* = 1027, 77%) did not undergo any form of curative treatment.

**Table 1:** Clinical disease characteristics at prostate cancer diagnosis

			All patients (*n* = 1473), *n* (%)
	Year of diagnosis		
		<2008	478 (32)
		2008–2011	309 (21)
		2012–2015	472 (32)
		2016–2019	214 (15)
	Age (yr) at diagnosis		
		Median (IQR)	73 (67–79)
		<50	7 (<1)
		50–60	100 (7)
		>6070	437 (30)
		>70–80	583 (40)
		>80	346 (23)
	PSA (ng/ml) at diagnosis		
		Median (IQR)	28 (11–100)
		<10	341 (24)
		10–20	271 (19)
		>20–50	310 (21)
		>50–100	165 (11)
		>100	360 (25)
		Unknown, *n*	26
	Gleason score		
		≤6	180 (15)
		7	414 (34)
		8–10	627 (51)
		Unknown, *n*	252
	T stage		
		T0	3 (<1)
		T1	307 (22)
		T2	448 (32)
		T3	532 (38)
		T4	113 (8)
		Unknown, *n*	70
	N stage		
		N0	196 (14)
		N1	164 (11)
		NX	1077 (75)
		Unknown, *n*	36
	M stage		
		M0	742 (51)
		M1	310 (22)
		MX	394 (27)
		Unknown, *n*	27
	D'Amico risk group classification		
		Low risk	68 (5)
		Intermediate risk	282 (20)
		High risk	687 (48)
		Metastatic disease	385 (27)
		Unknown, *n*	51
	Treatment approach		
		Curative treatment	315 (23)
		Noncurative treatment	1027 (77)
		Unknown, *n*	131

IQR = interquartile range; *n* = number of patients; PSA = prostate-specific antigen.

**Fig. 1:**
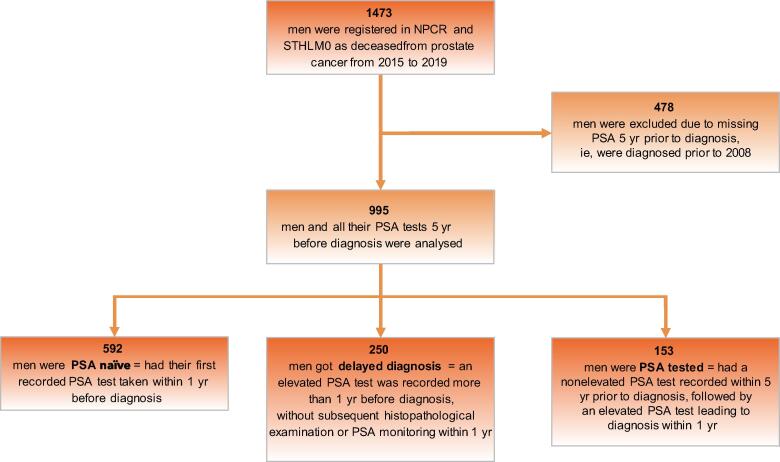
Flowchart illustrating the inclusion for the analysis of pre-diagnostic prostate-specific antigen testing patterns. NPCR = National Prostate Cancer Register; PSA = prostate-specific antigen; STHLM0 = Stockholm PSA and Biopsy Register.

**Fig. 2:**
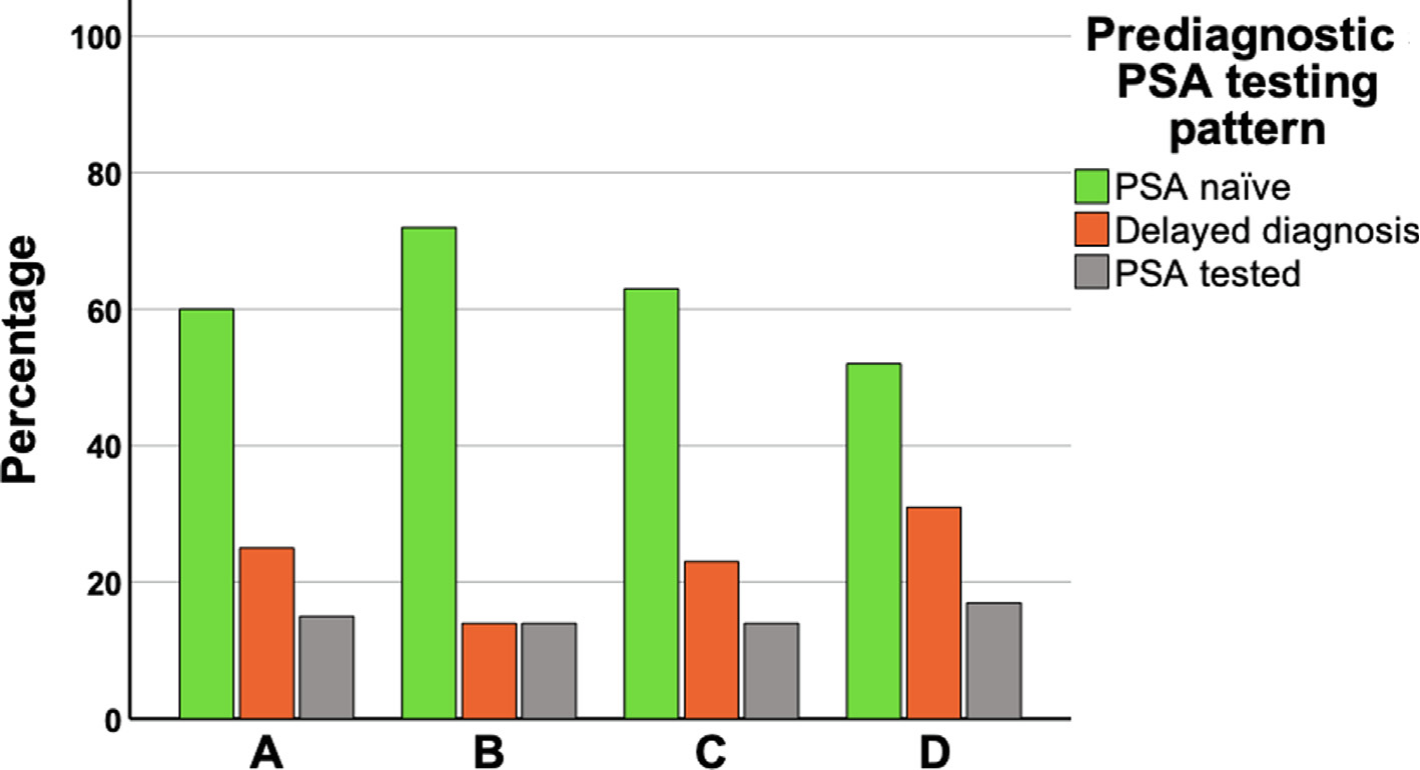
Prediagnostic prostate-specific antigen testing patterns, analysing (A) the entire cohort, and men with (B) age <65 yr at diagnosis, (C) age 65–74 yr at diagnosis, and (D) age ≥75 yr at diagnosis. PSA = prostate-specific antigen.

Figure 1 presents a flowchart illustrating the inclusion for the analysis of prediagnostic PSA testing patterns. As described, the STHLM0 database provided information regarding all PSA tests conducted after 2003, rendering it unfeasible to track individuals diagnosed before 2008 in the 5 yr leading up to their diagnosis. Consequently, 478 men were excluded from this analysis, leaving 995 men in the final cohort. A comparison between the excluded and analysed populations reveals higher disease severity in the analysed group. This is observed by their older age at diagnosis (48% compared with 28% diagnosed at age ≥75 yr), a greater proportion diagnosed with high-risk cancer (50% compared with 45%) and metastatic disease (34% compared with 12%), and reduced eligibility for curative treatment (21% compared with 30%). In the analysed cohort, Fig. 1, Fig. 2A reveal that 60% (*n* = 592) had not undergone PSA testing before diagnosis and were thus categorised as PSA naïve. Another 25% (*n* = 250) had undergone PSA testing with elevated results before diagnosis but lacked histopathological examination or a repeat PSA test within 1 yr, and were categorised as having delayed diagnosis. The remaining 15% (*n* = 153) had previously undergone PSA testing with nonelevated results but subsequently registered elevated PSA levels within 1 yr before their diagnosis, placing them in the PSA-tested category. Figures 2B–D illustrate the categories by age at diagnosis. A greater proportion of men were PSA naïve when diagnosed at a younger age, whereas for men diagnosed at an older age, delayed diagnosis was more prevalent. For patients categorised as having delayed diagnosis, there was a median delay time of 52 mo (IQR: 29–88 mo) between an elevated PSA test and subsequent diagnosis (Table 2). Notably, this delay time also depended on the age at diagnosis, with shorter delays observed among younger patients. Treatment approach had a curative intent (active treatment) in 18% of the PSA-naïve men, 23% of those categorised as having delayed diagnosis, and 29% of the PSA-tested men. In each group, the majority (82%, 77%, and 71%, respectively), did not undergo any form of curative treatment.

**Table 2:** Delay times for men categorised as having delayed diagnosis stratified by age groups and overall, and survival times stratified by age and risk groups and overall

			Age at diagnosis <65 yr (*n* = 22)	Age at diagnosis 65–74 yr (*n* = 82)	Age at diagnosis ≥75 yr (*n* = 146)	All men with delayed diagnosis (*n* = 250)
Delay times for men with delayed diagnosis stratified by age groups and overall						
	Delay time from detection of elevated PSA to diagnosis (mo), median (IQR)		27 (19–51)	51 (31–71)	63 (34–93)	52 (29–88)
			Age at diagnosis <65 yr (*n* = 291)	Age at diagnosis 65–74 yr (*n* = 572)	Age at diagnosis ≥75 yr (*n* = 610)	All patients (*n* = 1473)
Survival times stratified by age and risk groups and overall						
	Age at death (yr), median (IQR)		69 (65–73)	77 (74–83)	88 (83–91)	81 (74–87)
	Survival time after diagnosis (mo), median (IQR); *n* (%)		102 (48–152)	81 (34–146)	50 (23–100)	71 (30–132)
	Low risk		129 (109–172); 10 (4)	139 (107–166); 39 (7)	148 (94–181); 19 (3)	141 (105–169); 68 (5)
	Intermediate risk		135 (98–170); 64 (22)	124 (76–180); 127 (23)	98 (70–141); 91 (16)	115 (76–165);282 (20)
	High risk		98 (43–151); 121 (42)	74 (31–133); 224 (41)	52 (26–96); 342 (58)	65 (29–120); 687 (48)
	Metastatic disease		63 (36–130); 91 (32)	40 (19–75); 157 (29)	22 (10–38); 137 (23)	36 (17–70); 385 (27)

IQR = interquartile range; *n* = number of patients; PSA = prostate-specific antigen.

**Fig. 3:**
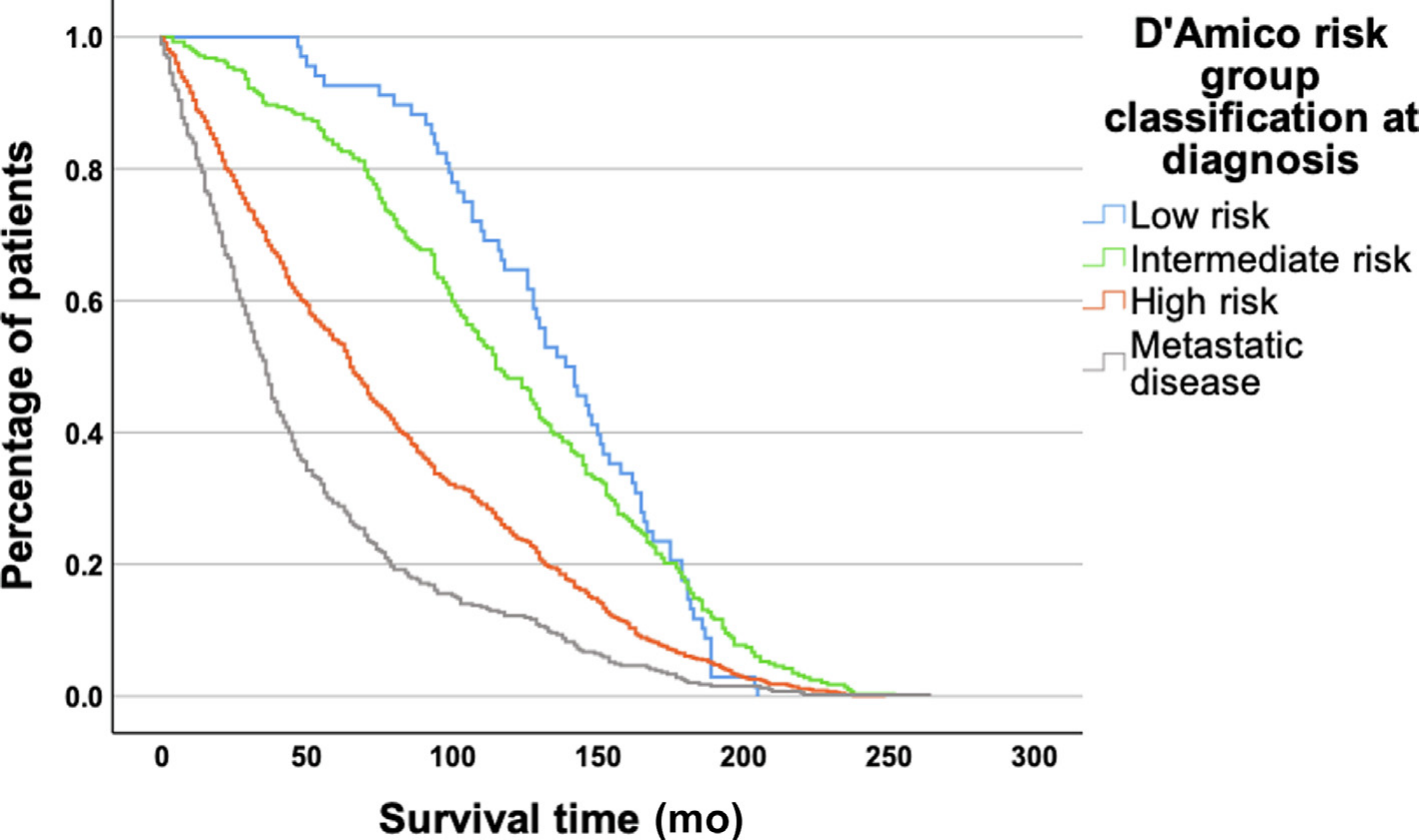
Time to death in patients who died from prostate cancer according to risk groups at diagnosis (*p* ≤ 0.001).

Table 2 presents survival times stratified by age and risk groups. The median age at death was 81 yr (IQR: 74–87 yr), and the median survival time after diagnosis was 71 mo (IQR: 30–132 mo). The median survival time after diagnosis was 141 mo (IQR: 105–169 mo) for patients diagnosed with low-risk cancer, 115 mo (IQR: 76–165 mo) for those with intermediate-risk cancer, and 65 mo (IQR: 29–120 mo) for those with high-risk cancer. Patients with metastatic disease at diagnosis had a median survival time of 36 mo (IQR: 17–70 mo). The survival times depended on age at diagnosis, with more prolonged survival in younger patients. The variations in survival among distinct risk groups are also illustrated by the curves in Figure 3, showing that low- and intermediate-risk cancer at diagnosis was significantly associated with longer survival from the time of diagnosis than high-risk cancer and metastatic disease (*p* < 0.001).

## 4 Discussion

In this population-based study of men who died from PC, our results reveal that a majority had not undergone PSA testing before diagnosis or were tested but not subsequently followed up, potentially causing a delay in diagnosis. Nearly half of the study population was diagnosed with high-risk cancer and almost one-third with metastatic disease. These findings suggest that many men who die from PC may be detected earlier in the natural history of the disease when treatments are more effective. This could implicate deficiencies in current opportunistic PSA testing strategies.

Our study shows that a significant number of men who die from PC have high-risk cancer or metastatic disease already at diagnosis and are mostly PSA naïve prior to diagnosis. Consequently, a majority get their diagnosis at an advanced stage when curative treatment intention is not applicable. Only a minor difference was observed among the prediagnostic PSA testing pattern groups. It is well documented that PC-specific mortality is high among men with advanced disease already at diagnosis [18]. While screening efforts have shown potential reductions in PC-specific mortality rates with as much as 40% observed in the Gothenburg segment of the large ERSPC trial [3], we must be cautious about concluding a mortality reduction of the population analysed in this study had they undergone screening. Such a conclusion would be influenced by several complicating factors well described by Berry [19], and these can be analogously applied to our context. First, our observations are confined to analysing a cohort exclusively comprising men who have died from PC. Second, differences in survival across various risk groups and age at diagnosis may be influenced by a lead time bias. A lead time bias refers to an overestimation of survival resulting from earlier detection through screening compared with clinical presentation. Considering the estimated lead time indicated in the ERSPC trial [20], we acknowledge that the lead time bias may exert a more pronounced influence on PC than other malignancies, given the natural course of the disease. Lead times also vary significantly when comparing a screened population with one exposed to opportunistic testing. This contrast is exemplified by the incidence of metastatic disease at diagnosis in a screened population compared with one exposed to opportunistic testing. In the ERSPC trial, the incidence of metastatic disease was slightly above 1% [4], whereas in our study population, it was almost one-third. However, since our study population comprised only opportunistically tested men deceased from PC and not both groups, determining the exact impact of the lead time bias on our findings was unfeasible. Nevertheless, our findings show that many men who die from PC receive their diagnosis at an advanced stage and highlight the need for further research on this topic.

PC diagnostics also extends beyond PSA testing. Prostate magnetic resonance imaging (MRI) has gained widespread adoption in recent years. It has been proved to reduce the detection of low-risk PC cases significantly when performed prior to prostate biopsy in men with elevated PSA levels, thus mitigating the risks of overdiagnosis and the adverse effects of PC screening [21], [22]. Other blood tests along with MRI can also supplement the PSA test in identifying men requiring biopsies, thereby reducing the overdiagnosis of insignificant PC cases [23], [24], [25], [26], [27], [28]. Current evaluations, such as the Organized Prostate Cancer Testing project, are underway in most regions of Sweden, seeking to implement and assess the value of organised testing that combines PSA, MRI, and biopsies using specific algorithms [29]. Further research and evaluation are essential to determine the effectiveness and significance of organised testing and its potential to reduce PC mortality without increasing the diagnosis of clinically insignificant cases.

This study is the first population-based study explicitly addressing opportunistic PSA testing in men deceased from PC. However, due to its retrospective and observational design, the study relies on existing data, which may, at times, contain omissions and incomplete information. For instance, we acknowledge that men with a family history of the disease face an increased risk of dying from PC, and this information was not assessed. Additionally, the availability of certain variables, such as specified T2 stage, was inconsistent, affecting our ability to assess disease severity and categorise patients accurately. We acknowledge that the D’Amico risk group classification tool proved suboptimal in this context. Notably, other tools offer superior risk stratification capabilities [30]. However, our focus was not to predict the mortality risk but to enable risk stratification among our patient cohort, thus warranting our selection of the D’Amico tool for its broad recognition.

Our results contain additional complexities. PSA test data were inaccessible before 2003, potentially leading to misclassification of patients who may have undergone PSA testing prior to that year but are erroneously categorised as PSA naïve. Within this group, there may also exist a small subset of men who have undergone PSA or pathology tests outside of Stockholm. In our effort to address this limitation, we followed the approach of Nordström et al [31], restricting our analysis to patients diagnosed after 2008, thereby extending the PSA test history to 5 yr. However, the reduced follow-up period from 2008 to 2019 prompts further investigation, due to the prolonged natural progression of PC. This relatively brief duration introduces a potential selection bias towards more aggressive PC cases, evident in the observed comparison of disease severity at diagnosis between the excluded and analysed populations. Thus, it is essential to emphasise that our results on prediagnostic PSA testing patterns offer observational findings, representing a subset of lethal PC cases and not the entire PC population. Concerning men categorised as having delayed diagnosis, it is also plausible that several valid reasons existed for the lack of monitoring, reasons that we cannot discern from the available dataset. Patients may have declined biopsies and opted not to follow their health care provider's recommendations. In some cases, particularly among older men, a deliberate decision may have been made to forgo further investigation. Finally, our study population is limited to men who died from PC in Stockholm County from 2015 to 2019. This county has been exposed to two prior screening trials throughout the follow-up period of our study, and the PSA tests recorded in STHLM0 lack information regarding whether they were conducted as part of a specific trial or opportunistically. Consequently, it is plausible that our study population is more extensively screened than the general PC population. If desired, this adds to the intrigue of our findings, wherein the majority was still identified as PSA naïve. Variations in testing intensity may also be influenced by geographic regions and socioeconomic factors, which were not explored in this study. Therefore, our findings may not comprehensively represent the entire demographic, health care, and diagnostic landscape of all men with lethal PC, impacting the generalisability of this study. In light of these limitations, caution is warranted when interpreting our results. Additional research involving larger and more diverse study populations, ideally including a control group, is crucial to confirm and build upon our findings.

## 5 Conclusions

We observed that many men with lethal PC exposed to opportunistic testing did not undergo PSA testing before diagnosis, or if they did, were not subsequently monitored. Nearly half of the study population was diagnosed with high-risk cancer and almost one-third with metastatic disease. Our findings align with prior evidence, emphasising potential deficiencies in the current opportunistic PSA testing approach. Additional studies are needed to evaluate the clinical impact on survival of these testing patterns and assess whether men with lethal PC could benefit from organised PSA testing or alternative screening initiatives.

  ***Author contributions*:** Markus Arvendell had full access to all the data in the study and takes responsibility for the integrity of the data and the accuracy of the data analysis.

  *Study concept and design*: Arvendell, Grönberg, Nordström, Lantz.

*Acquisition of data*: Arvendell, Björnebo, Eklund, Grönberg, Nordström.

*Analysis and interpretation of data*: Arvendell, Björnebo, Eklund, Grönberg, Nordström, Lantz.

*Drafting of the manuscript*: Arvendell.

*Critical revision of the manuscript for important intellectual content*: All authors.

*Statistical analysis*: Arvendell, Björnebo, Eklund.

*Obtaining funding*: None.

*Administrative, technical, or material support*: None.

*Supervision*: Nordström, Lantz.

*Other*: None.

  ***Financial disclosures:*** Markus Arvendell certifies that all conflicts of interest, including specific financial interests and relationships and affiliations relevant to the subject matter or materials discussed in the manuscript (eg, employment/affiliation, grants or funding, consultancies, honoraria, stock ownership or options, expert testimony, royalties, or patents filed, received, or pending), are the following: None.

  ***Funding/Support and role of the sponsor:*** None.
